# Supplementary material for: Causal Relationship between Aging and Anorexia Nervosa: A White-Matter-Microstructure-Mediated Mendelian Randomization Analysis
Source: Biomedicines. 2024 Aug 16;12(8):1874. doi: 10.3390/biomedicines12081874 (PMC11351342; doi:10.3390/biomedicines12081874)
Supplement: Supplementary file 1 [file biomedicines-12-01874-s001.zip › Supplementary Figure/Figure S4/Singe SNP plot of MR analysis for TL to ubm-b-1637.pdf]

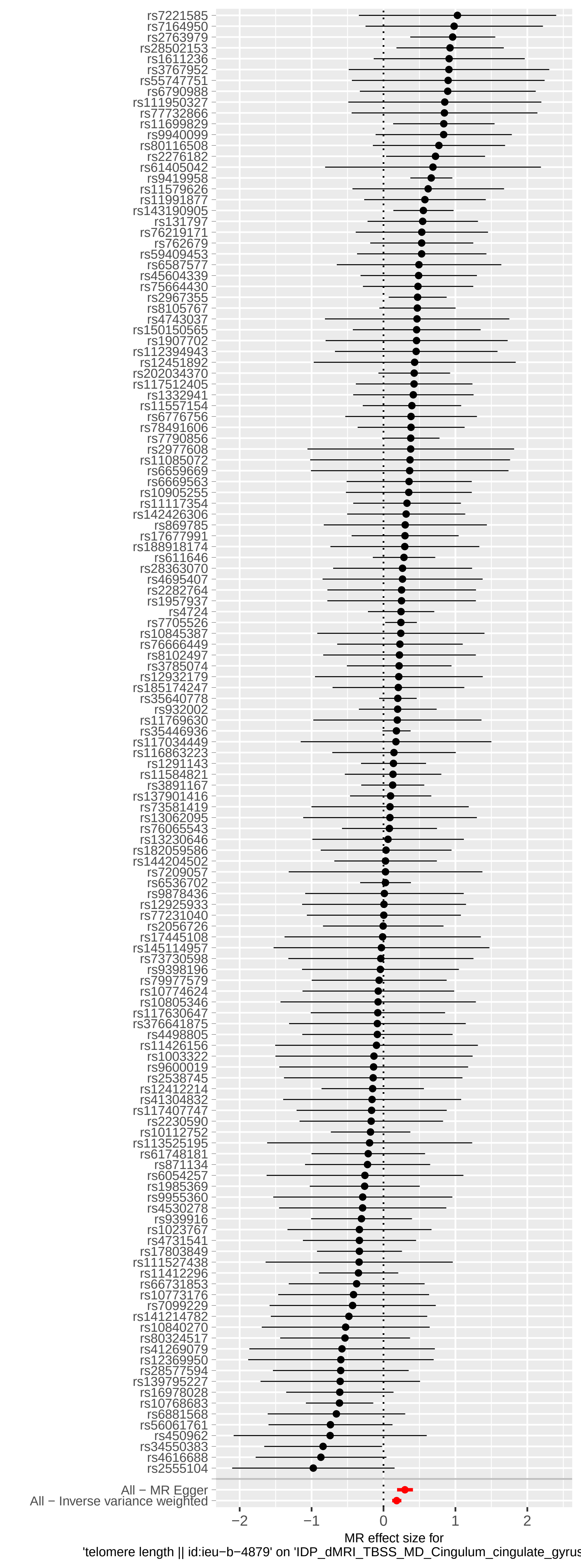
rs7221585
rs7164950
rs2763979
rs28502153
rs1611236
rs3767952
rs55747751
rs6790988
rs111950327
rs77732866
rs11699829
rs9940099
rs80116508
rs2276182
rs61405042
rs9419958
rs11579626
rs11991877
rs143190905
rs131797
rs76219171
rs762679
rs59409453
rs6587577
rs45604339
rs75664430
rs2967355
rs8105767
rs4743037
rs150150565
rs1907702
rs112394943
rs12451892
rs202034370
rs117512405
rs1332941
rs11557154
rs6776756
rs78491606
rs7790856
rs2977608
rs11085072
rs6659669
rs6669563
rs10905255
rs11117354
rs142426306
rs869785
rs17677991
rs188918174
rs611646
rs28363070
rs4695407
rs2282764
rs1957937
rs4724
rs7705526
rs10845387
rs76666449
rs8102497
rs3785074
rs12932179
rs185174247
rs35640778
rs932002
rs11769630
rs35446936
rs117034449
rs116863223
rs1291143
rs11584821
rs3891167
rs137901416
rs73581419
rs13062095
rs76065543
rs13230646
rs182059586
rs144204502
rs7209057
rs6536702
rs9878436
rs12925933
rs77231040
rs2056726
rs17445108
rs145114957
rs73730598
rs9398196
rs79977579
rs10774624
rs10805346
rs117630647
rs376641875
rs4498805
rs11426156
rs1003322
rs9600019
rs2538745
rs12412214
rs41304832
rs117407747
rs2230590
rs10112752
rs113525195
rs61748181
rs871134
rs6054257
rs1985369
rs9955360
rs4530278
rs939916
rs1023767
rs4731541
rs17803849
rs111527438
rs11412296
rs66731853
rs10773176
rs7099229
rs141214782
rs10840270
rs80324517
rs41269079
rs12369950
rs28577594
rs139795227
rs16978028
rs10768683
rs6881568
rs56061761
rs450962
rs34550383
rs4616688
rs2555104
All − MR Egger
All − Inverse variance weighted
−2
−1
0
1
2
MR effect size for
'telomere length || id:ieu−b−4879' on 'IDP_dMRI_TBSS_MD_Cingulum_cingulate_gyrus
